# Neuroprotective effect of a new synthetic aspirin-decursinol adduct in a rat model of ischemic stroke

**DOI:** 10.1186/cc12283

**Published:** 2013-03-19

**Authors:** YH Lee, CW Park, JH Cho, MH Won

**Affiliations:** 1 Kangwon National University, Chuncheonsi, South Korea

## Introduction

Stroke is a major cause of death. This study investigated the preventative effect of a new synthetic drug on brain function in experimentally induced ischemic stroke.

## Methods

Male Sprague-Dawley rats were administered aspirin (ASA), decursinol (DA) or ASA-DA before and after ischemic insults. Brain and neuronal damage were examined by TTC staining, PEP-CT, NeuN immunohistochemistry and F-J B histofluorescence. Gliosis was also observed by GFAP and iba-1 immunohistochemistry.

## Results

Pretreatment with 20 mg/kg, but not 10 mg/kg, of ASA-DA protected against ischemic neuronal death and damage, and its neuroprotective effect was much more pronounced than that of ASA or DA alone. In addition, treatment with 20 mg/kg ASA-DA reduced the ischemia-induced activation of astrocytes and microglia.

## Conclusion

Our findings indicate that ASA-DA, a new synthetic drug, prevents against transient focal cerebral ischemia, which provides a resource for the development of its clinical application for stroke.

